# The Homeodomain Transcription Factor NKX3.1 Modulates Bladder Outlet Obstruction Induced Fibrosis in Mice

**DOI:** 10.3389/fped.2019.00446

**Published:** 2019-11-12

**Authors:** Mehul S. Patel, Diana K. Bowen, Nicholas M. Tassone, Andrew D. Gould, Kirsten S. Kochan, Paula R. Firmiss, Natalie A. Kukulka, Megan Y. Devine, Belinda Li, Edward M. Gong, Robert W. Dettman

**Affiliations:** ^1^Department of Urology, Northwestern University Feinberg School of Medicine, Chicago, IL, United States; ^2^Gong Laboratory, Division of Pediatric Urology, Stanley Manne Children's Research Institute, Ann and Robert H. Lurie Children's Hospital of Chicago, Chicago, IL, United States; ^3^Department of Urology, Loyola University Medical Center, Maywood, IL, United States; ^4^Department of Pediatrics, Northwestern University Feinberg School of Medicine, Chicago, IL, United States

**Keywords:** Nkx3.1, bladder outlet obstruction, testosterone, androgen receptor, tissue fibrosis

## Abstract

Fibrosis is an irreversible remodeling process characterized by the deposition of collagen in the extracellular matrix of various organs through a variety of pathologies in children, leading to the stiffening of healthy tissues and organ dysfunction. Despite the prevalence of fibrotic disease in children, large gaps exist in our understanding of the mechanisms that lead to fibrosis, and there are currently no therapies to treat or reverse it. We previously observed that castration significantly reduces fibrosis in the bladders of male mice that have been partially obstructed. Here, we investigated if the expression of androgen response genes were altered in mouse bladders after partial bladder outlet obstruction (PO). Using a QPCR microarray and QRTPCR we found that PO was sufficient to increase expression of the androgen response gene *Nkx3.1*. Consistent with this was an increase in the expression of NKX3.1 protein. Immunofluorescent antibody localization demonstrated nuclear NKX3.1 in most bladder cells after PO. We tested if genetic deletion of *Nkx3.1* alters remodeling of the bladder wall after PO. After PO, *Nkx3.1*^*KO*/*KO*^ bladders underwent remodeling, demonstrating smaller bladder area, thickness, and bladder: body weight ratios than obstructed, wild type controls. Remarkably, *Nkx3.1*^*KO*/*KO*^ specifically affected histological parameters of fibrosis, including reduced collagen to muscle ratio. Loss of *Nkx3.1* altered collagen and smooth muscle cytoskeletal gene expression following PO which supported our histologic findings. Together these findings indicated that after PO, *Nkx3.1* expression is induced in the bladder and that it mediates important pathways that lead to tissue fibrosis. *As Nkx3.1* is an androgen response gene, our data suggest a possible mechanism by which fibrosis is mediated in male mice and opens the possibility of a molecular pathway mediated by NKX3.1 that could explain sexual dimorphism in bladder fibrosis.

## Introduction

Muscle hypertrophy and tissue fibrosis are common irreversible responses to obstruction seen in diseases affecting various organs such as the bladder, heart, blood vessels, lungs, and intestines. In part, this response is adaptive to allow the obstructed organ to overcome increased pressure. However, it becomes maladaptive over time with worsening fibrosis and organ dysfunction. There are significant gaps in our knowledge of the molecular and cellular processes that lead to muscle hypertrophy and fibrosis in obstructed systems. One emerging paradigm for fibrosis includes sexual dimorphism in which severity, depending on the type of fibrosis, varies based on sex. For example, in pulmonary arterial hypertension, females are more impacted than males (1). Fibrotic chronic kidney disease such as polycystic kidney disease, IgA nephropathy, and membranous glomerulopathy progress more slowly in pre-menopausal women than in age-matched men (2). Liver fibrosis associated with human immunodeficiency virus and hepatitis C virus coinfection is less severe in women than men (3). Other forms of liver injury and fibrosis through non-alcoholic fatty liver disease are known to have a predilection for men over women (4). Thus, sex is likely a key confounding factor in human fibrotic diseases.

Animal models have also implicated sexual dimorphism in the fibrotic process. For example, in a model that measured baboon intrauterine growth restriction, male fetuses responded to undernutrition with more cardiac fibrosis compared to females (5). In a sterile peritonitis model after oophorectomy in mice, loss of KLF11 reduced fibrosis in females with the response restored after administration of progesterone (6). Similarly, castration was found to be protective against fibrosis in male mice in the model, with testosterone replacement ultimately reversing this effect. Similar observations have been made in models of cardiac, kidney, and bladder fibrosis, indicating that sex alters tissue fibrosis in experimental animals (7–9).

In the urinary tract specifically, PO is a common urologic problem causing fibrosis that results from a number of pathologies. Posterior urethral valves (PUV) is a common congenital etiology in young boys that leads to bladder fibrosis with a prevalence of 2.1 per 10,000 live male births (10). Benign prostatic hyperplasia (BPH), which affects older men, is the most common cause of PO and leads to fibrotic remodeling of the bladder wall. Bladder remodeling can also happen in females after long-term bladder outlet obstruction secondary to pelvic organ prolapse (11). For BPH and pelvic organ prolapse, many treatment options are available, which prevents maladaptive remodeling from occurring if addressed early on. However, despite early treatment of the obstructing tissue in PUV, children often develop fibrotic bladders with poor function (12). Frequently, this progression worsens during early adolescence, suggesting that increased androgen levels during puberty could affect the development of fibrosis.

Mouse models of PO have demonstrated increased fibrosis with hypertrophy-thickened bladders (13). In a castrated PO model, however, bladders were noted to have an attenuated fibrotic response with preserved contractility (9). Upon testosterone replacement, the deleterious effects in the non-castrate model were ultimately restored, implicating testosterone as an essential contributor to maladaptive remodeling. Despite this connection, the mechanism of action by which androgens affect the bladder's response to obstruction is not well-understood. The androgen receptor (AR) pathway has been studied extensively and found to control a number of genes (14). We aimed to identify androgen response genes altered in the bladder specifically in PO and determine their function in bladder remodeling. We identified the AR response gene, *Nkx3.1*, as a gene upregulated after PO. Here, we present our results with genetic deletion of *Nkx3.1* in the context of PO in mice.

## Materials and Methods

### Experimental Animals

This study was carried out in accordance with the recommendations of surgical guidelines, Animal Care and Use Committee. The protocol was approved by the Institutional Animal Care and Use Committee of Northwestern University (protocol no. IS00003975). Initial experiments with DNA microarray and validation were performed on C57BL/6J mice (000664, Jackson). *Nkx3.1*^*KO*/*KO*^ mice were obtained from the laboratory of Dr. Sarki Abdulkadir and were reported to be in the C57BL/6 background. Generation of this mouse strain and primers used to genotype these mice are described in Abdulkadir et al. (15). Initial surgeries on *Nkx3.1*^*KO*/*KO*^ mice resulted in lethality near the time of recovery from surgery. This included mice subjected to sham laparotomy suggesting that lethality was a response to isoflurane anesthesia. Since we had not observed this either in C57BL/6 or CD-1 wild type mice, we reasoned that the *Nkx3.1*^*KO*/*KO*^ line may carry a secondary allele that rendered them susceptible to recovery from isoflurane anesthesia. To cross away this putative allele and to generate a genetic background similar to the one we used in Flum et al. (9) we backcrossed *Nkx3.1*^*KO*/*KO*^ mice to CD1 for three generations, and then bred back into the C57 BL/6 background for at least three generations. In each generation we selected mice both positive and negative for the *Nkx3.1*^*KO*^ allele by PCR. This allowed us to create two “backbred” lines that were in a highly similar genetic background: *Nkx3.1*^*KO*/+^ and wild type (*Nkx3.1*^+/+^). After each backcross, mice were subjected to sham surgery and it was determined if the lethal response to anesthesia was still present. Once it was determined that this response no longer remained, we interbred *Nkx3.1*^*KO*/+^ mice and selected for *Nkx3.1*^*KO*/*KO*^ males and females. Then these mice were interbred to establish the new *Nkx3.1* null line in the mixed CD1/C57 BL/6 background. Backbred wild type mice were used as controls in our study.

### Partial Bladder Outlet Obstruction Model

Male mice at 6–8 weeks of age were randomly assigned to sham operation, partial bladder outlet obstruction (PO), or surgical castration and partial bladder outlet obstruction (CPO). All surgeries were done as described previously described (9). Deviations from this protocol include the use of 6–0 Prolene around the bladder neck, 4–0 Vicryl for closure of the abdominal wall, and subcutaneous buprenorphine (0.05 mg/kg) for post-operative pain control. All mice were sacrificed at 1 or 4 weeks postoperatively by CO_2_ asphyxia followed by cervical dislocation. Bladders were dissected out at the level of the ligature, emptied of urine, and weighed prior to subsequent experiments.

### Void Stain on Paper (VSOP)

VSOP was performed and analyzed as previously described in Tassone et al. (16).

### RNA Expression QPCR Microarray

Bladders were harvested from mice 1 week after sham, PO, and CPO surgeries and placed in Trizol (Thermo Fisher). Tissue was homogenized in Trizol using a Bead Bug homogenizer and 3 mm zirconium beads. Total RNA was extracted from homogenized bladders using the RNeasy Plus Mini Kit. RNA was converted into cDNA using a High-Capacity cDNA kit (Applied Biosystems, Catalog #4368814). Differential expression was analyzed using an AR RT^2^ Profiler^TM^ Array (Qiagen 330231 PAMM-142ZA) to profile the expression of 84 genes that mediated signal transduction in cells responsive to male sex hormone.

### Quantitative PCR

Reverse transcriptase qPCR was performed as previously described (17). Validated primers were purchased from Applied Biosciences. Relative expression was quantified using the ΔΔCt method (18). In all cases GAPDH served as the internal control and expression under each condition was assessed relative to this gene. As ΔΔCt values are not paired, statistical significance was determined using ΔCt values, which are paired. Standard error of the mean was calculated using error propagation (19). This method takes into account experimental variation at each step in the analysis: Ct values, ΔCt values, and ΔΔCt values.

### Western Blot

Mouse bladders were homogenized in RIPA buffer (Thermo Fisher Scientific, Catalog #89900) containing 100X Halt Protease Cocktail Inhibitor (Thermo Fisher Scientific, Catalog #78440). The insoluble fraction was pelleted at 13,000 RPM for 20 min at 4°C. Soluble protein concentration was determined using the Pierce BCA Protein Assay Kit (Thermo Fisher Scientific, Catalog #23227) and absorbances were read using a BMG Labtech Clariostar microplate reader. Protein samples were diluted in 2X Laemmli sample buffer with 5% 2-mercaptoethanol. Proteins were resolved on Bolt™ 4–12% Bis-Tris Plus pre-cast gels. Gels were transferred using an iBlot 2 (Life Technologies) at 20 V for 7 min. Membranes were blocked and antibodies diluted in Tris buffered saline containing milk (5% w/v). Blots were developed using a Pierce™ ECL Western Blotting Substrate (Thermo Fisher Scientific, Cat: 34577) and imaged using a ChemiDoc MP Imager (BioRad). Densitometry was performed using ImageJ. The rabbit anti-mouse IgG, affinity purified antibody to NKX3.1 was from Athena Enzyme Systems (Catalog #0315).

### Histologic Analysis

Bladders were dissected at the base of the neck and urine expressed. Bladders were equilibrated in physiologic buffer (Dulbecco's PBS), fixed in 10% formalin for 24 h and sent to Northwestern's Mouse Histology and Phenotyping Laboratory for paraffin embedding, sectioning, and staining. Bladders were cut into 4 μm sections in the sagittal orientation inclusive of the bladder neck, dome, and body. They were stained with hematoxylin and eosin (H&E) as well as Masson's trichrome (MT). Sections were imaged at 5x and 40x magnification using a Leica upright microscope (DM R). Sections were randomized during the acquisition of the images. Bladder circumference, thickness, and area were computed using ImageJ (National Institutes of Health, Bethesda, MD, USA), and collagen: smooth muscle ratio and muscle content were quantified using Photoshop (Adobe) and averaged from 10 or more 40x images of each bladder. In the investigation of smooth muscle, regions of urothelium and stroma were excluded from the analysis. The method to use Photoshop to measure collagen accumulation as assessed by MT stain is described in detail in Dahab et al. (20). Here, red pixels (acid fuchsin and xylidine ponceau stained) are used to measure muscle in the detrusor and blue pixels (methyl blue stained) are used to measure fibrous collagen.

### Immunofluorescence Analysis

Bladders or prostates were dissected as above, equilibrated in physiologic buffer (Dulbecco's PBS) and fixed 12–18 h in 4% formaldehyde made from 16% paraformaldehyde in Dulbecco's PBS. Fixed tissue was washed in Dulbecco's PBS several times and equilibrated in sucrose (20% and 30% w/v). Tissue was placed in OCT compound (Tissue-Tek) and frozen on dry ice. Ten micrometer mid-coronal sections were cut and placed on Super Frost Plus glass slides (Thermo Fisher). Sections were blocked in Dulbecco's PBS with Tween-20 (0.1% v/v) and bovine serum albumin (0.1% w/v). Sections were probed with rabbit anti-mouse NKX3.1 (Athena Enzyme Systems), washed and then with donkey anti-rabbit IgG coupled with Alexa Fluor 568 (Thermo Fisher). Sections were counterstained with the 4′,6-diamidino-2-phenylindole (DAPI) nuclear stain. Sections were imaged on a Zeiss LSM 880 microscope using confocal optics.

### Statistical Analysis

Statistical analysis was performed using One- and Two-Way ANOVA (Prism 7.0e). Multiple comparisons with One- and Two-Way ANOVA followed Tukey Test parameters with family-wise significance and confidence level set to 0.05 (95% confidence interval).

## Results

### The Androgen Response Gene *NKX3.1* Is Upregulated in the Bladder After PO

**Figure 1:**
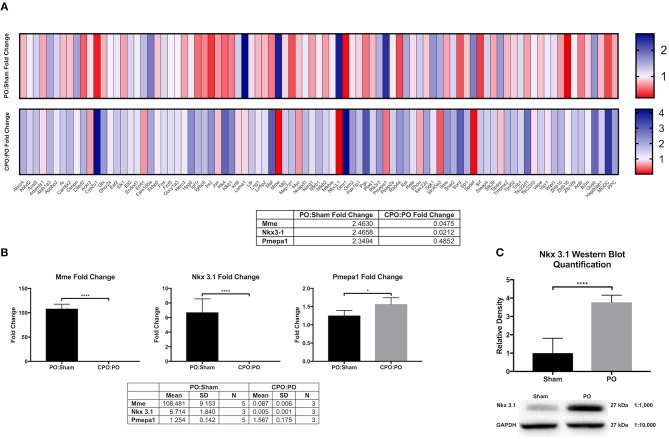
Bladder AR target gene analysis and validation. **(A)** Heat maps comparing androgen receptor target gene upregulation (blue) and downregulation (red) in PO:sham and CPO:PO groups (*N* = 3 for each surgical group). **(B)** RT-qPCR validation of the genes identified by the DNA microarray as upregulated in response to obstruction and downregulated in response to PO and CPO (*N* = 3 for each surgical group). **(C)** Western blot quantification of NKX3.1 in sham (*N* = 3) and PO (*N* = 3) bladders. Error bars in all graphs are represented as a standard deviation with family-wise significance and confidence level calculated at a 95% confidence interval (n.s., *P < 0.05 or ****P < 0.0001).

In hormonally intact mice, we observed that nuclear localized androgen receptor (AR) is increased in bladder cells suggesting that an androgen response is activated by PO (Firmiss and Gong, Supplementary Materials (doi: 10.6084/m9.figshare.9899312). Therefore, we hypothesized that AR response genes were transcriptionally altered following PO. To test this hypothesis, we interrogated a QPCR microarray of genes responsive to male sex hormone. We also tested a group of bladders taken from castrated male mice subjected to PO (CPO) to determine if change were affected by androgen levels. We specifically looked to identify genes that were increased after PO compared to sham and that were unchanged or downregulated in CPO compared to PO mice. Figure 1A shows the microarray heatmap demonstrating the transcriptional changes observed in PO and CPO mice. Here three genes met our criteria: *Mme*, which encodes neprilysin or membrane metallo-endopeptidase; *Nkx3.1*, which encodes NK3 homeobox 1 and *Pmepa1*, which encodes prostate membrane protein 1 androgen induced. The data for the QPCR array can be found here (doi: 10.6084/m9.figshare.9887408).

**Figure 2:**
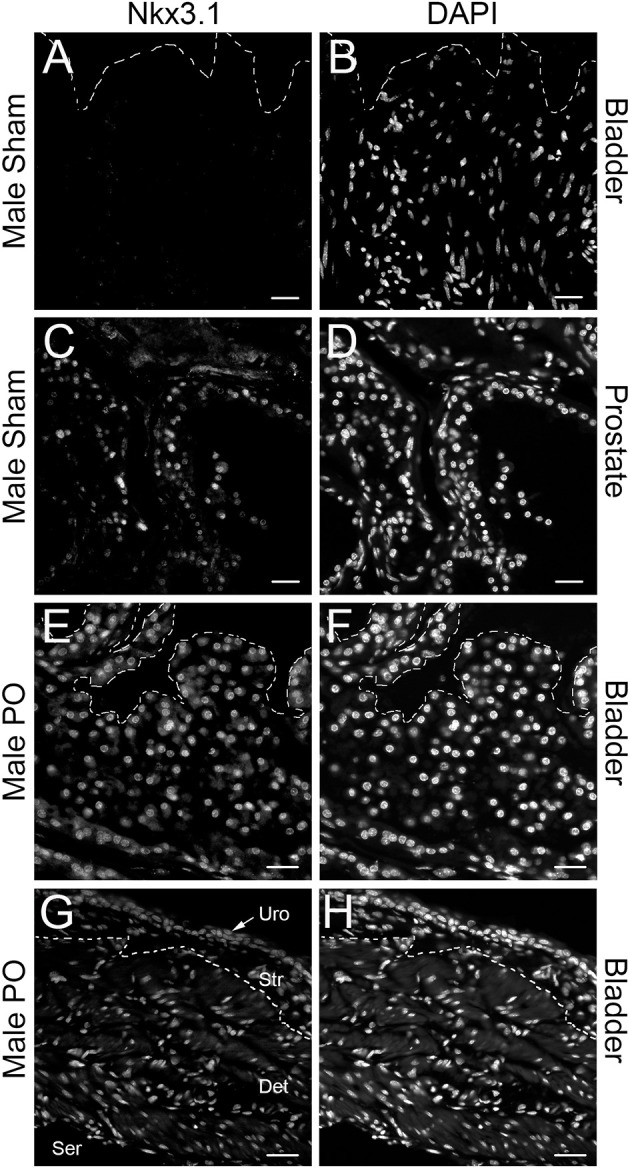
Immunolocalization of NKX3.1. Sham bladder stained with anti-NKX3.1 **(A)** and DAPI **(B)**; prostate from sham animal stained with anti-NKX3.1 **(C)** and DAPI **(D)**; PO bladder stained with anti-NKX3.1 **(E)** and DAPI **(F)**; separate PO bladder stained with anti-NKX3.1 **(E)** and DAPI **(F)** showing lower magnification with urothelium (Uro), stroma (Str), detrusor (Det) and serosal (Ser) layers. The orientation of each bladder is presented with the urothelium at the top of each panel and indicated by a dotted line in **(A,B,E,F)**. In **(G,H)** the dotted line marks the approximate border between stroma and detrusor. Magnification bars are 40 μm in **(A–F)** and 60 μm in **(G,H)**.

To validate these findings, we performed RT-qPCR in independently generated bladders (Figure 1B). Here we found that that the upregulation and downregulation was consistent for *Mme* and *Nkx3.1*, however, *Pmepa1* did not follow the same expression pattern. *Pmepa1* was found to be upregulated in both the PO and CPO models suggesting it may be activated in the setting of obstruction outside of the AR pathway. Meanwhile, both *Mme* and *Nkx3.1* seemed to be regulated in an androgen dependent fashion and, therefore, may function as regulators of bladder remodeling after obstruction. *Nkx3.1*^*KO*/*KO*^ mice were readily available for our use through partnership with the Abdulkadir Laboratory (Northwestern Feinberg School of Medicine, Department of Urology), and therefore, *Nkx3.1* was chosen as the gene of choice for further investigation. Prior to assessment of the effects of *Nkx3.1* deletion, we first attempted to further validate the finding of *Nkx3.1* expression in the bladder, as it was previously not reported to be active in this organ. We tested if the *Nkx3.1* protein product was increased after PO (Figure 1C). Here we observed minimal NKX3.1 protein in the sham group with much higher expression in the PO group. To localize this expression in bladders we stained sections from the sham operated and PO groups. Here we observed very little positive staining in bladders from the sham group (Figures 2A,B). Positive staining was observed in nuclei of prostate epithelial cells, indicating antibody activity on our sections (Figures 2C,D). Positive staining was observed in PO bladders (Figures 2E–H). This staining was nuclear and was surprisingly throughout the bladder. This included positive reaction in urothelium, stroma (Figure 2E) and detrusor muscle (Figures 2E,H). Thus, our results indicated that PO is sufficient to induce both *Nkx3.1* RNA and protein expression in the male mouse bladder. Localization of the protein indicated that multiple different cell-types induce nuclear expression of NKX3.1 in response to PO.

### Loss of Nkx3.1 Does Not Significantly Alter Bladder Development and Function

**Figure 3:**
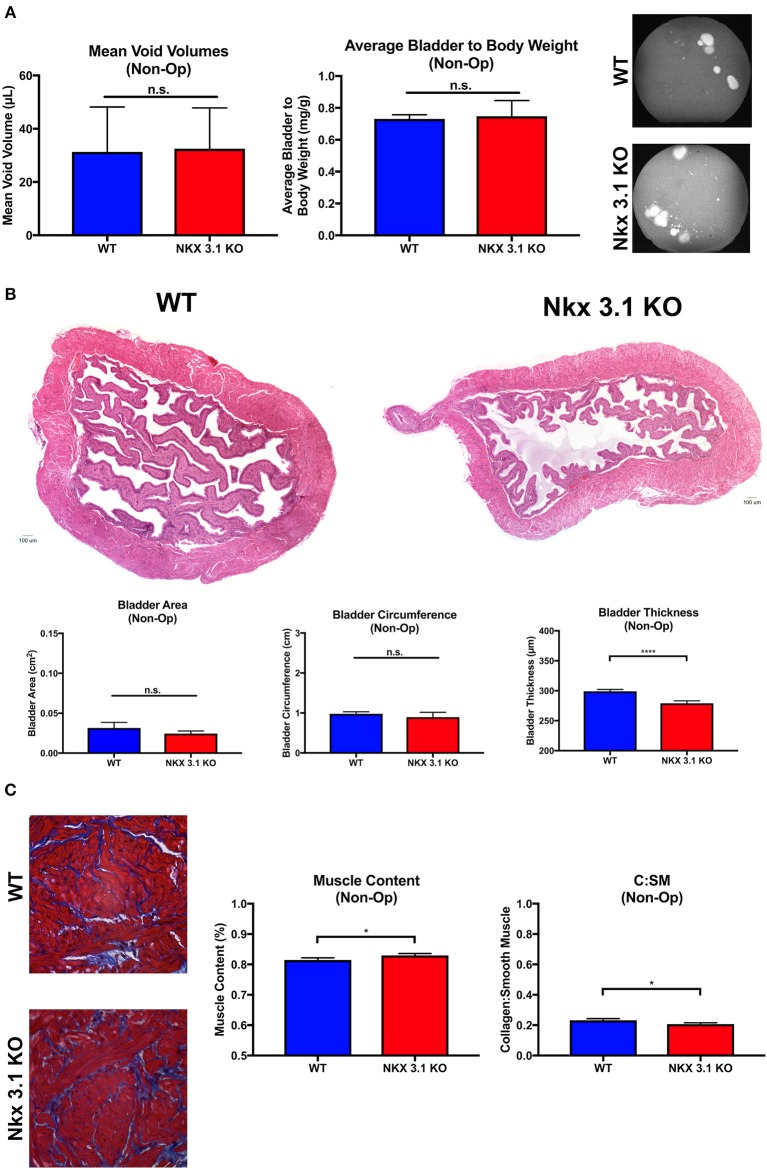
Functional and histologic analysis of unoperated WT and *Nkx3.1*^*KO*/*KO*^ bladders. **(A)** Comparison of mVV (WT, *N* = 4; *Nkx3.1*^*KO*/*KO*^, *N* = 4), bladder: body weight ratios (WT, *N* = 4; *Nkx3.1*^*KO*/*KO*^, *N* = 4), and representative VSOP images. **(B)** Histologic analysis of HandE images with basic bladder metrics such as bladder area, circumference, and thickness (WT, *N* = 4; *Nkx3.1*^*KO*/*KO*^, *N* = 4). **(C)** MT images, muscle content, and collagen: smooth muscle ratio (WT, *N* = 4; *Nkx3.1*^*KO*/*KO*^, *N* = 4). Error bars in all graphs are represented as a standard deviation with family-wise significance and confidence level calculated at a 95% confidence interval (n.s., *P < 0.05 or ****P < 0.0001).

To test if NKX3.1 has functions in the intact bladder at baseline, we analyzed bladder function and histology in *Nkx3.1*^*KO*/*KO*^ male mice. Previous studies have not implicated any role for the gene in the bladder (21). However, since we observed low levels of NKX3.1 protein in sham bladders (Figure 1C), there was the possibility that *Nkx3.1* bladder functions had been overlooked in previous studies. If present, such defects could alter our interpretation of bladder function after obstruction. Non-operated WT and *Nkx3.1*^*KO*/*KO*^ mice were assessed by voiding stain on paper (VSOP) and bladders were harvested after 4 weeks for weight. Figure 3A shows non-significantly different mean voided volumes (mVV) and bladder: body weight ratios for both groups. Similarly, bladder area and circumference were equal between WT and *Nkx3.1*^*KO*/*KO*^ mice (Figure 3B). One difference we found was that *Nkx3.1*^*KO*/*KO*^ bladders were 6.7% thinner at baseline (299.05 μm ± 3 vs. 279 ± 4 μm, *P* < 0.05). Another important indicator of normal bladder development is muscle content which is altered in the presence of obstruction. Bladders from non-operated WT and *Nkx3.1*^*KO*/*KO*^ mice were analyzed by MT staining (Figure 3C). Here we found that muscle content (81.52% ± 0.7% vs. 83.0% ± 0.6%, *P* < 0.05) was increased at baseline in *Nkx3.1*^*KO*/*KO*^ bladders. This was supported by the observation that collagen: smooth muscle ratio (0.2321 ± 0.011 vs. 0.207 ± 0.008, *P* < 0.05) was decreased in *Nkx3.1*^*KO*/*KO*^ bladders. Thus, loss of *Nkx3.1* alone had small but significant effects on structure indicating that NKX3.1 functions in the uninjured bladder.

### *Nkx3.1* Deletion Alters Bladder Remodeling After PO

**Figure 4:**
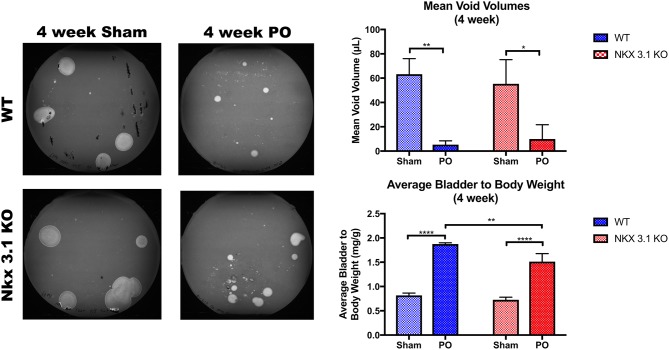
Functional analysis of WT and *Nkx3.1*^*KO*/*KO*^ mice after sham and PO. VSOPs demonstrated voiding patterns with mVV and bladder: body weight ratios for WT (Sham, *N* = 5; PO, *N* = 3) and *Nkx3.1*^*KO*/*KO*^ mice (Sham, *N* = 4; PO, *N* = 5). Error bars in all graphs are represented as a standard deviation with family-wise significance and confidence level calculated at a 95% confidence interval (n.s., *P < 0.05, **P < 0.01, or ****P < 0.0001).

**Figure 5:**
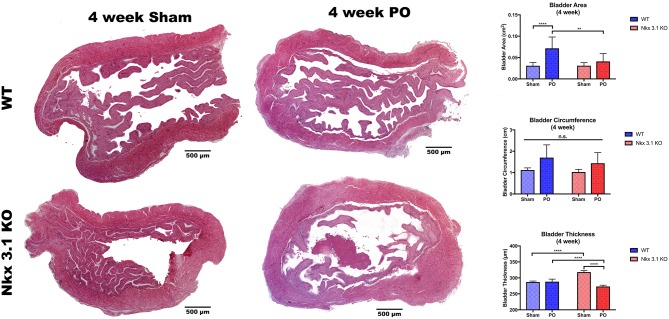
Morphologic analysis of WT and *Nkx3.1*^*KO*/*KO*^ mice after sham and PO. Representative whole bladder images and comparisons of bladder area, circumference, and thickness between WT (Sham, *N* = 5; PO, *N* = 3) and *Nkx3.1*^*KO*/*KO*^ (Sham, *N* = 4; PO, *N* = 5) mice. Error bars in all graphs are represented as a standard deviation with family-wise significance and confidence level calculated at a 95% confidence interval (n.s., **P < 0.01 or ****P < 0.0001).

**Figure 6:**
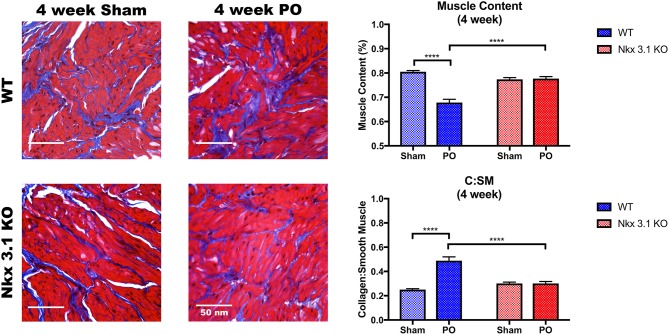
Histologic analysis comparing WT and *Nkx3.1*^*KO*/*KO*^ after sham and PO. Representative MT images and comparisons of muscle content and collagen: smooth muscle ratio between WT (Sham, *N* = 5; PO, *N* = 9) and *Nkx3.1*^*KO*/*KO*^ (Sham, *N* = 4; PO, *N* = 5) mice. Error bars in all graphs are represented as a standard deviation with family-wise significance and confidence level calculated at a 95% confidence interval (n.s., ****P < 0.0001).

To test if alterations to bladder remodeling after PO involve *Nkx3.1*, we investigated whether *Nkx3.1*^*KO*/*KO*^ mice undergoing PO could replicate our previous findings in castrated male mice. We compared functional and histologic data for *Nkx3.1*^*KO*/*KO*^ mice undergoing sham and PO surgeries. Figure 4 shows a lower mVV for *Nkx3.1*^*KO*/*KO*^ PO mice compared to *Nkx3.1*^*KO*/*KO*^ sham. Additionally, bladder: body weight ratios were significantly higher between PO and sham as expected. These findings are similar to the changes seen in WT after PO and sham, however, we found that bladder: body weight ratios for *Nkx3.1*^*KO*/*KO*^ PO were significantly lower than WT PO, indicating an altered remodeling process. Morphologically, *Nkx3.1*^*KO*/*KO*^ bladders had a similar area and circumference but significantly lower bladder thickness after PO compared to sham (Figure 5). This was a slightly different response than observed for WT, which showed a higher bladder area after PO compared to sham but non-significant changes in circumference and thickness. When examining collagen: smooth muscle ratio and muscle content, sham and PO bladders were also similar for *Nkx3.1*^*KO*/*KO*^ bladders, unlike the response seen in WT where collagen: smooth muscle ratio is increased and muscle content is decreased (Figure 6). Despite the absence of *Nkx3.1*, these bladders showed a response to obstruction. However, our data indicated that *Nkx3.1* null male bladders remodel differently than either hormonally intact WT or castrated mice. This supported the idea that *Nkx3.1* function represents a smaller process than is altered by castration in the context of bladder PO.

### *Nkx3.1* Deletion Alters Collagen and Smooth Muscle Gene Expression in the Bladder

**Figure 7:**
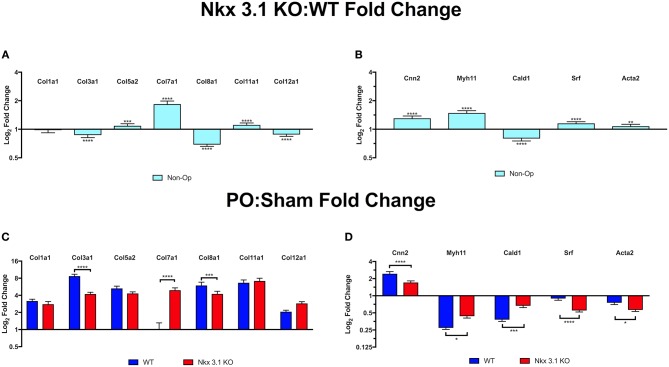
Quantitative PCR comparison of collagen and smooth muscle transcripts in WT and *Nkx3.1*^*KO*/*KO*^. **(A)** Collagen gene expression in non-operated *Nkx3.1*^*KO*/*KO*^ bladders (*N* > 3) relative to non-operated WT bladders (*N* > 3). Horizontal line indicates point where the relative level of expression is the same in *Nkx3.1*^*KO*/*KO*^ and WT bladders. Bars above the line indicate increased expression in *Nkx3.1*^*KO*/*KO*^. **(B)** Smooth muscle gene expression in non-operated *Nkx3.1*^*KO*/*KO*^ bladders (*N* > 3) relative to WT (*N* > 3). **(C)** Collagen gene expression in sham operated (blue bars) and PO operated (red bars) *Nkx3.1*^*KO*/*KO*^ bladders relative to WT. **(D)** Smooth muscle gene expression in sham operated (blue bars) and PO operated (red bars) *Nkx3.1*^*KO*/*KO*^ bladders (*N* > 3) relative to WT (*N* > 3). In all graphs horizontal lines indicate a value of 1 which is no change in gene expression. Values above the line indicate increased gene expression in *Nkx3.1*^*KO*/*KO*^ bladders. Values below the line indicate decreased gene expression in *Nkx3.1*^*KO*/*KO*^ bladders. The Y axis indicates log_2_ fold change as calculated using the ΔΔCt method. The Y axis is presented using a logarithmic scale. Error bars in all graphs are represented as a standard deviation with family-wise significance and confidence level calculated at a 95% confidence interval (n.s., *P < 0.05, **P < 0.01, ***P < 0.001, or ****P < 0.0001).

To investigate how NKX3.1 affects collagen matrix deposition and loss of muscle, we studied expression of collagen and smooth muscle genes in non-operated, sham and PO bladders. We expected there to be no difference in collagen gene expression in non-operated bladders comparing *Nkx3.1*^*KO*/*KO*^ and WT. However, here we found that multiple collagens were slightly altered in expression; most notably, *Col7*α*1* had significantly increased expression in *Nkx3.1*^*KO*/*KO*^ mice at baseline compared to WT (Figure 7A). This was surprising because it demonstrated that Nkx3.1 regulates collagen gene expression in male adult bladders. When comparing collagen gene expression after PO relative to sham, WT bladders demonstrated an increase in all collagens, except *Col7*α*1*, while *Nkx3.1*^*KO*/*KO*^ bladders showed an increase in each collagen evaluated (Figure 7C). When comparing the increases in collagen gene expression between PO and Sham, WT showed a significantly higher difference compared to KO for two collagens (*Col3*α*1* and *Col8*α*1*), consistent with our prior histologic findings of greater fibrosis in WT bladders after PO. Interestingly, *Col7*α*1* followed a different pattern. WT bladders showed an equivalent expression of *Col7*α*1* after PO and sham. However, KO bladders showed a significant induction in *Col7*α*1* expression after PO compared to sham. While *Nkx3.1* seems to globally affect the transcriptional response of certain collagens, it seems to be particularly important as a negative regulator of *Col7*α*1* at baseline and in response to obstruction.

Loss of *Nkx3.1* appeared to preserve muscle in PO bladders based on MT stain. We therefore tested if smooth muscle gene expression was maintained in *Nkx3.1*^*KO*/*KO*^ bladders after PO. In non-operated mice, all smooth muscle genes other than *Cald1* are slightly increased in expression at baseline in *Nkx3.1*^*KO*/*KO*^ compared WT, although these differences are not as pronounced as the upregulation in *Col7*α*1* (Figure 7B). Given our previous findings of preserved muscle histologically in *Nkx3.1*^*KO*/*KO*^ bladders after PO, we expected *Nkx3.1*^*KO*/*KO*^ bladders to have preserved smooth muscle gene expression compared to WT. When comparing smooth muscle gene expression after PO relative to sham, both WT and *Nkx3.1*^*KO*/*KO*^ bladders had decreased levels of all muscle markers except for *Cnn2*, which increased (Figure 7D). While WT bladders showed a significantly greater decrease in *Myh11* and *Cald1* expression, *Nkx3.1*^*KO*/*KO*^ bladders showed a significantly greater decrease in *Srf* and *Acta2*. Although these findings are not what we initially expected, it indicates that perhaps the differences in muscle content percentages between WT and *Nkx3.1*^*KO*/*KO*^ are in fact a result of the differences in collagen content.

## Discussion

Although little research has focused on the effects of androgens on the bladder itself rather than the effects of the prostate on the bladder outlet, it has been previously suggested that there is a correlation between androgen activity and bladder dysfunction (22). Little is known, however, about the functional importance of the androgen pathway in bladder physiology after embryonic development, specifically in the setting of outlet obstruction. Our lab has previously shown that castrate, partially-obstructed male mice who undergo testosterone replacement have higher bladder weights and higher collagen: smooth muscle ratios than intact animals or those without testosterone replacement (9). Although the exact mechanism of action of the androgen pathway on the bladder is yet to be determined, it is clear that it does indeed play a role.

To further underscore this, our results have now identified *Nkx3.1*, as a critical mediator of the fibrotic response. Future experiments such as demonstrating that AR binds to the *Nkx3.1* promoter in response to PO will need to be done to cement the role of *Nkx3.1* as a direct response to AR. Nonetheless, our work here shows that partial bladder obstruction is sufficient to induce *Nkx3.1* expression in the bladder and that loss of *Nkx3.1* has significant effects on the remodeling that occurs after PO. Functionally, however, these bladders were similar to WT in mVV, in contrast to our previous findings with castration. As *Nkx3.1* is only one of a large number of genes involved in the intricate androgen pathway, it is likely there are additional pathways responsible for the other effects noted in the castration model.

*Nkx3.1* was initially found as an androgen regulated homeobox gene located mainly in the prostate, and was extensively studied as a tumor suppressor in prostate cancer (23, 24). Multiple studies, however, have since linked it to other tissue types and downregulation to their associated malignancies including the breast, oral mucosa, and liver (25–28). There is scarce literature describing *Nkx3.1* expression specifically in the bladder. In fact, NKX3.1 positivity has been shown to be a useful adjunct marker in differentiating prostate and urothelial carcinomas (29, 30). At baseline, we observed low levels of *Nkx3.1* expression in the bladder and surprisingly an effect on *Col7*α*1* expression in the knockout. This indicates that *Nkx3.1* may indeed have some functions in the normal bladder. Remarkably this effect was on a collagen gene that has not been implicated as a target of NKX3.1. This and our other findings involving collagen genes merit further study into how NKX3.1 regulates these genes as well as its involvement in modifying collagen architecture.

Importantly, bladder development and function are preserved in *Nkx3.1* null mice. In the prostate, *Nkx3.1* deletion has been shown to result in defects in prostate ductal morphogenesis and secretory protein production (21). Although our results cannot exclude a role for *Nkx3.1* in bladder development, they do demonstrate that the bladder is able to develop with normal voiding function and histologic features in its absence with small but significant differences in collagen: smooth muscle ratio, and bladder thickness.

Despite identifying a downstream androgen receptor target in bladder remodeling after obstruction, it remains unclear how NKX3.1 ultimately affects this process. The overall response to injury from PO is likely a multifactorial process, with NKX3.1 playing a small but critical role. NKX3.1 was shown to have an interactive relationship with the Myc oncoprotein (31). In fact, the authors of this study proposed that NKX3.1 and Myc both bind to a number of target genes and had opposing effects on the expression of these genes. In this model, NKX3.1 and Myc had dynamic and potentially opposing functions. Myc has been implicated in regulating smooth muscle cell proliferation and survival (32, 33). Thus, NKX3.1 could be induced in the bladder in response to obstruction to modify pathways controlled by the Myc oncoprotein.

*Nkx3.1* loss has previously been shown to be related to increased expression of VEGF-C in prostate cancer (34). If NKX3.1 represses VEGF-C in obstruction, we might expect to see alterations to blood vessels. Deletion of *Nkx3.1* in the context of PO would lead to increased neoangiogenesis which may improve healing and remodeling after obstruction. pBOO has been shown to cause tissue hypoxia and upregulation of the HIF1 pathway and subsequent studies have shown that inhibition of this pathway with 17-Dimethylaminoethylamino-17-demethoxygeldanamycin (17-DMAG) after PO improved bladder function and collagen: smooth muscle ratio (35–37). Although these results seem to contradict our findings, VEGF remained relatively similar in their PO + placebo and PO + 17-DMAG group, even though they showed improvement in bladder remodeling and voiding function. This may indicate that perhaps the benefits of blocking the HIF1 pathway are from alternative pathways outlined by Iguchi et al. (38).

Additionally, *Nkx3.1* may mediate its function through regulation of stem cell differentiation. The murine bladder has previously been shown to support a population of mesenchymal stem cells, which *Nkx3.1* may modulate (39). MicroRNAs have been shown to have a critical role in keratinocyte differentiation through silencing of *Nkx3.1* (40). Similarly, *Nkx3.1* may play a critical role in bladder remodeling by inhibiting stem cell response to obstruction. Future studies will attempt to clarify the stem cell response to PO and the role of *Nkx3.1* in this process.

PO is a common urologic pathology seen in young boys with PUV, males with BPH, and females with pelvic organ prolapse. The long-term effects of obstruction on the bladder are irreversible and, therefore, early treatment is imperative. Despite prompt treatment in PUV, these patients often progress to poorly functioning fibrotic bladders. Identifying the molecular mechanisms of this process will provide potential therapeutic targets to prevent this maladaptive remodeling process from occurring. Our findings identify *Nkx3.1* as a key modulator of fibrosis. As *Nkx3.1* has mainly been studied in the realm of prostate cancer, it's role in organ fibrosis has not previously been described. As it clearly plays a role in bladder fibrosis, its effect on other organs and fibrotic remodeling will warrant further investigation. The clinical implications of our study are that there is likely a molecular basis for differences in bladder fibrosis observed between males and females and that pharmacologic approaches to modulate NKX3.1 in prostate cancer could be extended to treat obstructive uropathies.

## Conclusion

We have identified *Nkx3.1* as an important mediator of bladder fibrosis in bladder outlet obstruction. Although the bladder seems to be morphologically and functionally intact, fibrosis is attenuated in the absence of *Nkx3.1*. Maladaptive changes to collagen and smooth muscle specific gene expression after PO were ameliorated by loss of *Nkx3.1*. Our results provide evidence that *Nkx3.1* plays a role in both bladder homeostasis and fibrosis.

## Data Availability Statement

The raw data supporting the conclusions of this manuscript will be made available by the authors, without undue reservation, to any qualified researcher.

## Ethics Statement

This study was carried out in accordance with the recommendations of surgical guidelines, Animal Care and Use Committee. The protocol was approved by the Institutional Animal Care and Use Committee of Northwestern University (protocol no. IS00003975).

## Author Contributions

MP, DB, and NT contributed to data collection, analysis, conception, and design. MP and RD wrote the first draft of the manuscript. NT performed the statistical analysis. AG, KK, PF, NK, MD, and BL contributed to data collection and analysis. EG and RD contributed to analysis, conception, and design. MP, NT, AG, EG, and RD contributed to manuscript revisions, read, and approved the submitted version.

### Conflict of Interest

The authors declare that the research was conducted in the absence of any commercial or financial relationships that could be construed as a potential conflict of interest.

## Supplementary Material

Supplemental Table 1 Raw data from AR target gene qRT-PCR microarray. Control group is RNA from sham operated bladders. Group 1 is RNA from PO operated bladders. Position is the position on the microarray plate. Symbol is the gene abbreviation. AVG Ct is the average of three biological replicates. Standard deviation is the standard deviation of the biological replicates.

Supplemental Table 2 Raw data from AR target gene qRT-PCR microarray. Control group is RNA from PO operated bladders. Group 1 is RNA from CPO (PO in castrated mice) operated bladders. Position is the position on the microarray plate. Symbol is the gene abbreviation. AVG Ct is the average of three biological replicates. Standard deviation is the standard deviation of the biological replicates.

Supplemental Table 3 RT2 Profiler PCR Array Key. This is the key to the plates for which data is shown in Supplemental Tables 1, 2.

Supplemental Figure 1 Accumulation of nuclear localized androgen receptor after partial bladder outlet obstruction. Top: Bar graph showing quantification of nuclear localized AR in the bladder after (left to right) sham, partial obstruction in a normal male mouse (PO), partial obstruction in an orchiectomized mouse (CPO) partial obstruction in an orchiectomized mouse administered testosterone (CPOT) and partial obstruction in an orchiectomized mouse administered dihydrotestosterone (CPOD). Percent nuclear stain indicates the percent of red pixels (AR) that are colocalized with blue (DAPI) pixels. Sample sizes are: sham (5), PO (5), CPO (6), CPOT (4), CPOD (4). Error bars indicate standard error of the mean. Significance is indicated by lines extending between bars. Bottom are representative images used during the quantification. Blue represents DAPI nuclear stain and red indicates staining with anti-androgen receptor antibody.

## Abbreviations

PO: partial bladder outlet obstruction
CPO: partial bladder outlet obstruction in a castrated male mouse
AR: androgen receptor
PUV: posterior urethral valves
BPH: benign prostatic hyperplasia
VSOP: voiding stain on paper
H&E: hematoxylin and eosin staining
MT: Masson's trichrome staining
ANOVA: analysis of variance
WT: wild type
mVV: mean voided volumes.
