# Supplementary figures and images for: The Homeodomain Transcription Factor NKX3.1 Modulates Bladder Outlet Obstruction Induced Fibrosis in Mice
Source: Front Pediatr. 2019 Nov 12;7:446. doi: 10.3389/fped.2019.00446 (PMC6861332; doi:10.3389/fped.2019.00446)

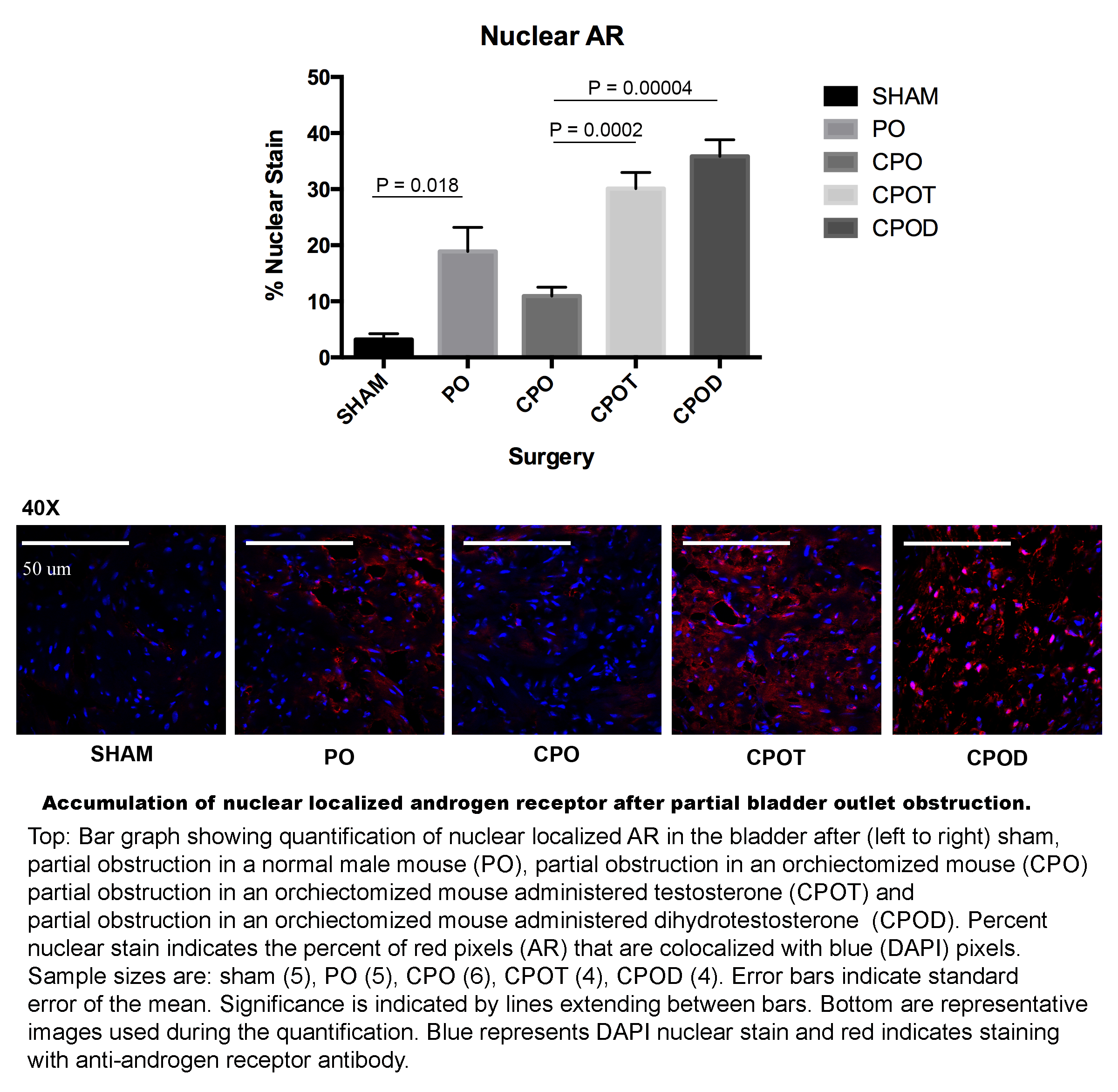

Supplement: Supplemental Figure 1 — Accumulation of nuclear localized androgen receptor after partial bladder outlet obstruction. Top: Bar graph showing quantification of nuclear localized AR in the bladder after (left to right) sham, partial obstruction in a normal male mouse (PO), partial obstruction in an orchiectomized mouse (CPO) partial obstruction in an orchiectomized mouse administered testosterone (CPOT) and partial obstruction in an orchiectomized mouse administered dihydrotestosterone (CPOD). Percent nuclear stain indicates the percent of red pixels (AR) that are colocalized with blue (DAPI) pixels. Sample sizes are: sham (5), PO (5), CPO (6), CPOT (4), CPOD (4). Error bars indicate standard error of the mean. Significance is indicated by lines extending between bars. Bottom are representative images used during the quantification. Blue represents DAPI nuclear stain and red indicates staining with anti-androgen receptor antibody. [file Image_1.tif]
